# Clinical engineering and uncertainty in clinical measurements

**DOI:** 10.1007/s13246-014-0288-3

**Published:** 2014-07-19

**Authors:** Emmanuel Koumoundouros

**Affiliations:** Department Electrical and Electronic Engineering, Melbourne School of Engineering, The University of Melbourne, Melbourne, VIC Australia

A number of qualifications can lead to employment in Clinical Engineering. A Clinical Engineer (CE) can be an Engineer who has completed an accredited Engineering degree with three years of relevant experience, an Engineering Associate who holds an Advanced Diploma in technical or nursing fields, or an Engineering Technologist who has completed a recognised Science/Technology degree that can work within clinics and/or provide specialised services for clinics [1–4]. One generic skill of the CE is the understanding and interpretation of physical and physiological measurements and the immediate recognition of physiological waveforms displayed on a bedside monitor and indentify uncharacteristic behaviour of these signals. The CE is well aware of the uncertainty of clinical measurements and with the advancement of technology and medical IT, one can considers the history of individuals and incorporate this information into the variance of the measurements. As an Associate Editor, it is not uncommon to see manuscripts sent in with measurements presented with over six significant figures for large scale values, the default of the analytical package, when, at best, the error allows for no decimal place. This carelessness can mislead readers about the accuracy of the methodology and/or analysis. As Engineers and Scientists, we need to be vigilant of how measurements are used and consider that variability of a measured quantity not just its mean.

The CE is trained to consider both instrumentation (technical) variance and biological (physiological) variance in measurements. Some aspects to consider are:

(i) in instrumentation, the calibration of a parameter is acceptable within a narrow 99 % confidence interval (CI);
(ii) in Biology and Physiology, the acceptable range is nominally the 95 % CI; and
(iii) all measurements should be within the range of biological and physiological expectations.

Uncertainties are physical limitations of the accuracy of measuring devices. Accuracy is the proximity of the measurement to the true value or the reliability of the measurement within an uncertainty. Each device has the precision listed in the specification and precision, the repeatability, or reproducibility of the measurement? The precision of a device should be seen in the number of significant digits the device displays. It would be expected that the more significant digits that a device displays, the more precise the instrument. However, this cannot automatically be assumed to be true and should be tested.

A critical consideration of the uncertainty in measurement is that the biological or physiological quantity being measured has an uncertainty that is not dependent on the device’s measurement limitation. This uncertainty depends upon the distribution of the repeated measurements of the subject being monitored. The accuracy is the device’s ability to measure the TRUE value of the physical entity it is measuring. In this editorial we consider three frequently monitored physiological measurements: the Electrocardiogram (ECG); the Blood Pressure; and Pulse Oximetery. Examine their uncertainty in measurement and conclude with a general application of uncertainty to clinical measurements.

An ECG monitor can measure body surface potentials down to 0.2 mV with a time resolution of 1 ms, but only displays the waveform to 1 cm = 1 mV resolution; it records the derived and averaged Heart Rate (HR) to ±1 beat per minute (bpm) with a running average computed [5]. The accuracy allows the HR parameter of the device to go to three significant places. Here, we see that the device displaying this measurement was designed for the purpose for which it is used. No clinicians will change their course of action just because the HR has gone from 75.432 to 75. 433.

With the advent of Heart Rate Variability (HRV), one can use the accuracy of the ECG monitor to determine HRV with a clinical acceptable mean of 141 ± 39 ms (mean and standard deviation) for a healthy subject. This equates to the 95 % confidence of short term variance of time between two consecutive heart beats in healthy adults being 104 ms [6–8]. Therefore, if we were to take the average HR of the 75 bpm and apply the 95 % CI based on average healthy HRV range of 65–85 bpm this make the average HR of healthy individuals 75 ± 10 bpm. Surely, this is a more accurate and reproducible representation of the true HR, where the device accuracy is used to derive HRV, which is the uncertainty of this measurement that is defined by one individual’s physiological variance and this variance adds to the therapeutic value of the ECG.

Another common and essential clinical measurement is the assessment of blood pressure. A bedside, physiological, non-invasive monitoring device measuring the cuff blood pressure may be a purely mechanical (pneumatic) device or an automated electromechanical device. The basic mechanical device has variances purely based on the physical size of the instrument, where a small dial that measures the pressure has a direct influence on the resolution of the measurement. This measurement is not only affected by instrumentation uncertainty but is also dependent on subjective uncertainty. There are spurious errors of the observer hearing the onset and decay of the Korotkoff sounds or observing the start and end of oscillations on the needle of the aneroid dial. There is also variance in the design control of the device. The instrumentation uncertainty is governed by AS ES 1060.3:2004 (manual) and AS/ANS3200.2.30:2001 (automated), where the accuracy of data is stated as a maximum of ±5 mmHg, with a standard deviation of 8 mmHg over 20 repeated measurements [9, 10]. This equates to allowing a 95 % CI of uncertainty in the accuracy of the measurement to under or over estimating the reading by the mean of 5 ± 15 mmHg and the pressures are reported with the accuracy of single units. In the lower range case of this uncertainty, a systolic pressure of 130 mmHg can have a 95 % CI range of 120 to 140 mmHg. This makes the systolic pressure reading with a 95 % CI 130 ± 10 mmHg, the instrumentation displays measures down to 1 mmHg and the uncertainty can be up to 20 mmHg (the upper range of the 95 % CI uncertainty). Clinicians should be aware of this wide uncertainty and re-consider whether cuff pressure changes say below 10 mmHg are an actual change in pressure.

The more accurate method of measuring blood pressure is the invasive or direct blood pressure device. According to AS/NZS 3200.2.34:1996, the approval and test specification of Medical electrical equipment, the particular requirements for safety of direct blood-pressure monitoring equipment requires an operating range for the pressure transducer to be within a static pressure of 4,000 and −400 mmHg. This standard states that the dynamic pressure at 1 Hz that is reflected in manufacturer specification that the uncertainty of the pressure measurement, be within 4 mmHg, [5]. This means the 95 % CI for a systolic pressure of 130 mmHg is 126–134 mmHg, but as in HR case, we need to account for the variability of beat to beat systolic pressure and this variance could be up to 30 % and is dependent on the volumetric state of an anaesthetised or subdued patient that varies from 6 to 18 % [11, 12]. This makes the physiological variance with a 95 % CI range from 122–138 to 107–153 mmHg depending on volumetric state. It is the range of this variance that gives volumetric state of the patient and the current devices average this out. It is essential here that we are all aware of, and have accounted for, the dynamics of the catheter-transducer system and have the system critically damped and does not over or under estimate the pressure.

Pulse Oxymetery has become the standard, most commonly used, clinical performance indicator of both the circulatory and respiratory systems. The Oxymeter detects the deflected/observed light and analyses the wavelengths of oxygenated and de-oxygenated blood to derive a saturation percentage. This saturation is then mapped to partial pressure of Oxygen in the peripheral arteries. At arterial oxygen saturations above 90 %, the variability between true and recorded saturation is 5 % and, therefore, the 95 % CI for a saturation reading of 95 % is 90–100 %. Below 90 % saturation, when the attention of the clinician is required, the 95 % CI goes up by 10 % [13]. This range is where a small drop in the saturation levels can make a critical difference in terms of patient management of hypoxia due to the characteristics of the saturation curve and delivering O2 to the tissues. Also, other factors can alter the accuracy of Pulse Oxymetery, such as skin pigmentation, location of the sensor, and ambient light. The more reliable way to measure partial pressures of gas in blood is to take blood samples.

The above examples of ECG, the Blood Pressure and the Pulse Oxymetery are the basic and commonly used clinical measured parameters, These parameters are time variable and the technique for measurement, as illustrated, introduces variability and bias, and all devices have their calibration issues. So we expect a measurement to be of the form.

$${\text{Y}}\_{\text{measured }} = {\text{ Y}}\_{\text{true }} + {\text{ Delta}}\_{\text{technique}} + {\text{ U}}\_{\text{device}}$$

where the measurement (Y_measured) is the sum of the true measurement (Y_true), the error introduced by the technique of measurement and the uncertainty specific to the device (+ U_device). Typically U_device is bounded, typically determined by the calibration. The Delta_technique is typically modelled as a random variable, with a bias and a variance error, which is independent of the device but dependent on biology/physiology, and can be considered stationary over a short period of measurement. The length of this period can be within weeks for healthy individuals or within minutes for critically cared individuals.

Then we re-consider the above equation as time variant.

$${\text{Y}}\_{\text{measured }}\left( {\text{t}} \right) \, = {\text{ T}}\left( {\text{s}} \right){\text{ y}}\_{\text{true }}\left( {\text{t}} \right) \, + {\text{ Delta}}\_{\text{technique}} + {\text{ U}}\_{\text{device}}$$

where T(s) has history of individuals and T(s){−1} may not exist in general, and hence the sampling period and sampling interval becomes very important. In which case we have a Least Squares approximation or algorithmic error in the recovery of the y_true. We report on a Y_estimate based on Y_measured, and the error between y_true and y_estimate includes all the errors of technique, device, but also the inclusion of measurement algorithm errors [14].

The accuracy of a device is its ability to measure the true level of the physiological parameter. The repeatability of accuracy is a measure of its ability to indicate the same value of the measured quantity with repeated measurements. With scientific instruments, poor repeatability can be a sign of poor accuracy, but with medical devices, as illustrated above, poor repeatability is also a sign of wide variance that is inherent in biological systems. Here, the two terms of accuracy and uncertainty are independent and one cannot be replaced by the other. To achieve better measurement, we aim to be more accurate with less uncertainty. Here, the CE ensures, with the annual preventative maintenance schedule, that the measurements of the device are well within the specifications of the manufacturer. The manufacturer’s design control processes produce the precision within the device that has the instrumentation uncertainty of the measurement well within the physiological variance. Accuracy is measured against a gold standard of measurement and the accuracy of a particular instrument is how close the instrument indicates the true value of the quantity. Medical devices have a high accuracy and repeatability but also have a high physiological variance that is dependent on the physiological state of the individual patient. This variance should be indicated by the measuring devices so that it can be properly interpreted and understood by the clinicians.

The CE faces a number of uncertainties in clinical measurement. The CE has the skill, along with the clinicians, to understand those variances and uncertainties. The CE can also respond appropriately to the variance in measurement and technically maintain the certainty in the measurements of all devices. This skill is just one of the many skills that the CE professional must develop, and a vital service that is offered to the Health Care Industry that is not offered by third party services on a day-to-day basis. 
